# The comparative diagnostic efficacy of BNP & NT-proBNP in Chronic kidney disease patients with complications

**DOI:** 10.12669/pjms.41.7.11826

**Published:** 2025-07

**Authors:** Saif Ullah Shaikh, Ghazala Yasmeen, Rehana Rehman, Abdul Manan Junejo

**Affiliations:** 1 Saif Ullah Shaikh, MBBS, MSc, MPhil, PhD Student. Department of Physiology, University of Karachi, Karachi, Pakistan; 2 Ghazala Yasmeen, MSc, PhD. Assistant Professor, Department of Physiology, University of Karachi, Karachi, Pakistan; 3 Rehana Rehman, MBBS, M.Phil., PhD, FHEA, MHPE. Professor & Director Graduate Studies, Department of Biological & Biomedical Sciences, Aga Khan University, Karachi Pakistan; 4 Abdul Manan Junejo, MBBS, FCPS, FRCP (Glasgow), Professor & Chairman, Department of Nephrology, Jinnah Post graduate Medical Center (JPMC), Karachi, Pakistan

**Keywords:** B-type Natriuretic Peptide, Chronic Kidney Disease, Cardiovascular Risk, Glomerular Filtration Rate, N-terminal pro-B-type Natriuretic Peptide

## Abstract

**Objective:**

To study the differences between the B-type Natriuretic Peptide (BNP) and the N-terminal pro-B-type Natriuretic Peptide (NT-proBNP) values in CKD patients due to diabetes and hypertension and without CKD. Another objective was to to observe what correlation the variables of age showed with the parameters of BNP, NT-proBNP values, and with Glomerular Filtration Rate (GFR).

**Methods:**

A case-control study was performed for six months between January to June 2024. In total 254 individuals took part in the study, with ages between 30-75 years, of which 85 were healthy controls and 169 CKD cases. CKD patients were recruited from the Department of Nephrology Jinnah Postgraduate Medical Centre (JPMC), Karachi. Diagnosis was made on the basis of raised serum urea, creatinine levels, and electrolyte imbalance. The following were excluded from participation: individuals with coronary artery disease, pregnancy, more than twice-weekly dialysis, or on steroid therapy. CKD cases were also staged according to National Kidney Foundation GFR guidelines.

**Results:**

Among the <50-year-old participants, BNP was abnormal in 92.7% of patients (p = 0.001), while NT-proBNP was abnormal in 38.5% (p = 0.01). When BNP and NT-proBNP were compared between the same individuals, BNP detected more abnormal cases, with a statistically significant difference (p = 0.01), indicating its better diagnostic yield in this group.

**Conclusion:**

BNP was identified as a more authentic marker than NT-proBNP in order to detect cardiovascular stress in CKD patients. Its close correlation with increasing age and decreasing GFR highlights its prognostic significance and potential role in routine cardiovascular risk assessment in CKD populations.

## INTRODUCTION

Chronic Kidney Disease (CKD) is a persistent loss of kidney function or structural change for more than three months. CKD occurs in an estimated 8–16% of the world’s population and is often neglected in its initial stages.^1^ When considering United States population alone, around 37 million individuals are living with CKD, and a further 20 million are at risk.^2^ In Pakistan, prevalence rates among adults vary from 12.5% to 29.9%, which places it as a major contributor to the healthcare burden in the country.^3^

Cardiovascular disease (CVD) is the most fatal complication of chronic kidney disease (CKD) and accounts for a major portion of the burden of illness and mortality in this patient population.^4^ CKD patients are two- to four-fold more likely to develop cardiovascular complications like cardiac failure, arrhythmias, peripheral artery disease, coronary artery disease (CAD), and stroke.^5^ This heightened susceptibility is further exacerbated by prevalent comorbidities for instance hypertension, diabetes mellitus, dyslipidemia, obesity and smoking. Even with this strong correlation, cardiovascular risk in patients with CKD tends to be under-recognized and undertreated.^6^

In light of these challenges, the discovery of valid, noninvasive biomarkers to forecast cardiovascular events in CKD is a high-priority clinical need.^7^ Of these most promising of markers, BNP and NT-proBNP are two which are released by the cardiac ventricles when there is myocardial wall stress and volume overload.^8^ BNP is active biologically and has been a popular mode of diagnosis of cardiac failure. Its inactive byproduct, NT-proBNP, is more stable, has a longer half-life, and hence is a marker of choice in the majority of diagnostic conditions.^9^

Some researchers have investigated the prognostic significance of natriuretic peptides in CKD patients. Miyakuni et al. and Wan et al. showed that levels of NT-proBNP are independently linked to higher cardiovascular mortality in patients with decreased Glomerular Filtration Rate (GFR), even without apparent cardiac disease.^10^,^11^ Other research, however, implies that BNP can offer greater diagnostic sensitivity in advanced CKD because it has a more direct representation of acute cardiac stress.^12^

Although they are clinically useful, both BNP and NT-proBNP levels have been shown to increase as renal function worsens, especially at GFR values of less than 15 mL/min/1.73 m².^13^ This overlap makes interpretation difficult and requires careful comparison between the two markers at each CKD stage to establish their relative utility.

This research, thus, seeks to compare BNP and NT-proBNP levels in CKD patients secondary to diabetes and hypertension and healthy controls. It also seeks to investigate the correlation of these biomarkers with age and renal function (as measured by GFR), to assess their diagnostic value across CKD stages.

## METHODS

A six months case-control study was conducted between January and June 2024 among individuals with CKD secondary to diabetes mellitus and hypertension. Participants were chosen from the nephrology outpatient department of Jinnah Postgraduate Medical Centre (JPMC), Karachi. Patients initially enrolled at JPMC were subsequently monitored and assessed at CBC Healthcare (Reference No: CBC/HC.PH II/No. 61, Date January 1, 2024) located in DHA Phase II, Karachi.

**Table-I:** Age (years) with Glomerular Filtration Rate, Brain Natriuretic Peptide and N-Terminal Pro- Brain Natriuretic Peptide.

		Age (years)		
		< 50 years (n=109)	50-75 years (n=60)	P-value
GFR	Stage 3A Moderate	18	2	0.005
		16.5%	3.3%	
	Stage 3B Moderate	17	4	
		15.6%	6.7%	
	Stage 4 Severe	74	54	
		67.9%	90.0%	
BNP	Normal	8	4	0.001
		7.3%	6.7%	
	Abnormal	101	56	
		92.7%	93.3%	
Pro BNP	Normal	67	48	0.013
		61.5%	80.0%	
	Abnormal	42	12	
		38.5%	20.0%	
		100.0%	100.0%	

Where GFR Glomerular filteration rate BNP Brain natriuretic peptide; ProBNP Pro brain natriuretic peptide *Chi-square test and Fischer Exact test.

### Ethjical Approal:

It was provided by the inistitutional review board, Ethics Committee of JPMC (Approval No: F.2-81/2024-GENL/54/JPMC, Date: July 29, 2024).

We included 254 participants, including 169 CKD cases and 85 healthy controls. The sample size was estimated using OpenEpi Version 3.0 with a 95% confidence interval, power of 80%, and 5% margin of error to ensure adequate statistical power to identify differences in biomarker levels between the groups.^14^

Controls were adults 30–65 years with normal renal function and no known systemic disease. CKD patients were of comparable age range with diabetic or hypertensive nephropathy for a minimum of five years. Exclusionary criteria were coronary artery disease, pregnancy, dialysis patients more than twice weekly, those on peritoneal dialysis or post-transplant, and subjects on steroidal or hormonal treatment. An informed consent in writing was given by all subjects, and confidentiality was ensured.

Data was collected using a standardized questionnaire encompassing demographic information, medical history, blood pressure, comorbid conditions, and habits. Laboratory testing involved hemoglobin, serum urea, creatinine, electrolytes, and Glomerular Filtration Rate (GFR), and B-type Natriuretic Peptide (BNP) and N-terminal pro-B-type Natriuretic Peptide (NT-proBNP) levels. Staging of CKD was according to estimated GFR derived from the MDRD 4-variable equation, with classification into stages 1 to 5 per the NKF-KDOQI guidelines.

Venous blood samples (6 mL) were drawn at 8:00 a.m. after overnight fasting in EDTA tubes, centrifuged for 10 minutes at 3,500 rpm at 4°C, and plasma was frozen at −90°C. Concentrations of BNP and NT-proBNP were analyzed using ELISA kits (BNP: Cat # ELK 4892; NT-proBNP: Cat # ELK 5423), while other biochemical parameters were measured on the Abbott AXSYM Microparticle Enzyme Immunoassay system.

## RESULTS

The study sample included 254 participants, 169 patients with chronic kidney disease (CKD) and 85 controls who were healthy. The mean age was considerably greater in CKD patients than the control group (p < 0.0001). In the CKD group, age had a marked correlation with estimated glomerular filtration rate (GFR), as well as with serum concentrations of B-type natriuretic peptide (BNP) and N-terminal pro-B-type natriuretic peptide (NT-proBNP). Subjects younger than 50 years were more frequently located in moderate CKD stages, while subjects aged between 50 and 75 years were more often classified in advanced stages of the disease (p = 0.005). Both age groups had elevated BNP and NT-proBNP levels; however, values outside the normal range were disproportionately found in younger patients (p = 0.001 and p = 0.013, respectively).

A considerably noteworthy discrepancy was found in comparing the two cardiac biomarkers between CKD cases. While a few patients had normal levels for both BNP and NT-proBNP, most had elevated BNP levels with NT-proBNP levels within the normal range (p = 0.01). This indicates a possible difference in the diagnostic sensitivity or response pattern between the two markers.

## DISCUSSION

This research compared BNP and NT-proBNP levels in patients with CKD due to diabetes and hypertension and healthy controls, and investigated their correlation with age and GFR. Our findings show that BNP levels were raised in a great number of CKD patients in both age groups, with abnormal values in 92.7% of patients <50 years and 93.3% aged 50–75 years (p = 0.001). Contrary to this, NT-proBNP was elevated in just 38.5% and 20.0% of patients in the corresponding age groups (p = 0.013). This supports the fact that BNP is a more sensitive biomarker than NT-proBNP in identifying cardiovascular risk among CKD patients.

Cardiovascular complications take the lead when diminished survival in CKD patients is assessed, with death being more frequent from cardiac conditions.^15^,^16^ This increased risk is fueled by a number of underlying mechanisms, such as chronic inflammation, calcium and phosphate imbalance regulation, oxidative stress, and the formation of vascular calcifications.^17^ B-type natriuretic peptide (BNP), a hormone secreted by the heart in response to elevated wall tension and pressure, is a biochemical marker of these hemodynamic alterations. In our research, high BNP levels in almost all CKD patients support its clinical usefulness in tracking cardiovascular stress.

**Table-II:** Correlation between Brain Natriuretic Peptide & Pro-Brain Natriuretic Peptide levels in chronic kidney cases.

BNP Normal Range in all ages (≤ 100pg/ml)	Pro BNP Normal Range in all ages ≤ 125 pg/ml)		Total	P-value
	Normal Cases	Abnormal Cases		
Normal cases	12(7.10%)	0(0%)	12(7.10%)	0.01
Abnormal cases	103(60.90%)	54 (32.0%)	157(92.9%)	
Total	115(68.0%)	54(32.0%)	169(100%)	

*Fischer exact test.

The noted correlation between decreased GFR and advancing age in CKD patients (p = 0.005) is in agreement with earlier findings from an Ontario cohort, wherein 27.6% of patients aged 60 years or more had GFR levels less than 60 mL/min.^18^ Considering the limited resources in most healthcare facilities, focusing CKD screening on high-risk groups, especially older adults with advanced cardiovascular disease, has been suggested as a cost-saving strategy.^19^,^20^

Our results also indicate a discrepancy between BNP and NT-proBNP measurements: although BNP was often raised, NT-proBNP was within normal limits in more than half the patients. One explanation for this discrepancy may be the differing physiological characteristics of the two peptides. BNP is biologically active and more directly sensitive to acute myocardial wall stress changes, while NT-proBNP, a biologically inactive fragment with a longer half-life, is more affected by renal clearance than by cardiac load.^13^ Thus, in patients with compromised kidney function but without associated cardiac strain, NT-proBNP can build up due to decreased clearance rather than active secretion, confining its specificity.^21^

These results are corroborated by previous research. A 2023 clinical study reaffirmed markedly high BNP concentrations in CKD patients and suggested its application as a cardiovascular prognostic marker.^22^ In contrast, Takahama et al. presented a conflicting report that mentioned NT-proBNP levels were raised in CKD even without apparent cardiac pathology but mostly due to impaired renal clearance.^21^ In the present study, BNP was more effective than NT-proBNP in detecting cardiovascular stress and hence is a better biomarker for this CKD group. However, this also points to the importance of interpreting levels of natriuretic peptide with caution, keeping in mind the renal status underlying.

Variability in NT-proBNP elevation with CKD etiology has also been reported in international studies. A Japanese study showed more robust associations between CKD progression and NT-proBNP levels in non-diabetic subjects as opposed to diabetic nephropathy.^23^ Our findings disagree, as we found lower sensitivity of NT-proBNP in a population dominated by diabetic and hypertensive nephropathies. These differences may be due to genetic, lifestyle, or ethnic influences on biomarker excretion and metabolism.

Other variables that affect BNP and NT-proBNP are age, obesity, gender, and comorbid conditions.^24^ For example, obesity will downregulate BNP levels, whereas ageing will independently elevate NT-proBNP levels. Even a recent European study implicated that the BNP/NT-proBNP ratio could possibly predict future cardiac remodeling, including left ventricular hypertrophy, suggesting that the two markers would have complementary applications in clinical practice.^25^

### Strength of the study:

The strength of this research is its clinically relevant emphasis on CKD patients secondary to two of the world’s most prevalent comorbidities, diabetes and hypertension and its concurrent comparison of BNP and NT-proBNP by CKD stage.

### Limitations:

Nevertheless, some limitations must be mentioned. Firstly, the study was conducted at a single institution with a relatively limited sample size, which may diminish the generalizability of the findings to more vast populations. Second, echocardiographic assessment of cardiac function was not performed to verify structural or functional cardiac modifications consistent with elevation of biomarkers. Third, possible confounding factors like BMI, smoking habits, and long-standing hypertension duration were not specifically analyzed for a relation with levels of BNP and NT-proBNP separately.

## CONCLUSION

This research shows that BNP is a more sensitive and effective marker than NT-proBNP for evaluating cardiovascular risk in patients with CKD. Due to its greater correlation with disease severity and age, BNP can be useful in early diagnosis and management of CVD in CKD patients. Routine inclusion of BNP testing in evaluation protocols could enhance prognostic predictability and clinical outcomes.

### Recommandations

Based on the findings of this study, future research should focus on conducting large scale, longitudinal and multicentered studies to validate the diagnostic and prognostic utility of BNP in patients with CKD across different stages and clinical subgroups. Moreover, studies should consider genetic, ethnic and metabolic differences that may influence biomarker expression and interpretation.

### Author’s Contribution:

**SUS:** Conception, design, and interpretation of data, drafting and revision of the article.

**GY:** Acquisition of data, analysis and interpretation, drafting and revision of the article.

**RR:** Interpretation of data & revision of the article.

**MJ:** Interpretation of data, contributed to patient access, scrutiny, monitoring and critical revision.

All authors have approved final approval version to be published and are accountable for all aspects of the work.
